# Aging-related episodic memory decline: are emotions the key?

**DOI:** 10.3389/fnbeh.2013.00002

**Published:** 2013-02-01

**Authors:** Kiyoka Kinugawa, Sophie Schumm, Monica Pollina, Marion Depre, Carolin Jungbluth, Mohamed Doulazmi, Claude Sebban, Armin Zlomuzica, Reinhard Pietrowsky, Bettina Pause, Jean Mariani, Ekrem Dere

**Affiliations:** ^1^Neurobiologie des Processus Adaptatifs, UMR 7102, Université Pierre et Marie Curie, Paris 6Paris, France; ^2^CNRS, UMR 7102Paris, France; ^3^Institut de la longévité, AP-HP Hôpital Charles Foix, Ivry-sur-SeineParis, France; ^4^Institute of Experimental Psychology, University of DüsseldorfDüsseldorf, Germany; ^5^Center for the Study and Treatment of Mental Health, Ruhr-Universität BochumBochum, Germany

**Keywords:** aging, anxiety, episodic memory, depression, working memory

## Abstract

Episodic memory refers to the recollection of personal experiences that contain information on what has happened and also where and when these events took place. Episodic memory function is extremely sensitive to cerebral aging and neurodegerative diseases. We examined episodic memory performance with a novel test in young (*N* = 17, age: 21–45), middle-aged (*N* = 16, age: 48–62) and aged but otherwise healthy participants (*N* = 8, age: 71–83) along with measurements of trait and state anxiety. As expected we found significantly impaired episodic memory performance in the aged group as compared to the young group. The aged group also showed impaired working memory performance as well as significantly decreased levels of trait anxiety. No significant correlation between the total episodic memory and trait or state anxiety scores was found. The present results show an age-dependent episodic memory decline along with lower trait anxiety in the aged group. Yet, it still remains to be determined whether this difference in anxiety is related to the impaired episodic memory performance in the aged group.

## Introduction

Episodic memory refers to the conscious recollection of a personal experience that contains information on what has happened and also where and when it happened (Tulving, 1983). Remembering a personal experience might also be accompanied by the remembrance of specific perceptions, emotions, and thoughts one had during a particular experience (Tulving, 2002). Episodic memory deficits are observed after medial temporal lobe injury (Nyberg et al., 1996) which includes important memory structures such as the hippocampus (Burgess et al., 2002) and amygdala (Markowitsch and Staniloiu, 2011) but also after lesions to the frontal cortex (Kirchhoff et al., 2000) and diencephalic structures, such as the mediodorsal thalamus and the mammillary bodies (Tsivilis et al., 2008; Wolff et al., 2008).

Episodic memory function is also extremely sensitive to cerebral aging (Shing et al., 2010; Nyberg et al., 2012), neurodegenerative (Williams-Gray et al., 2006; Dubois et al., 2007), and psychiatric diseases (Dere et al., 2010). Cross-sectional studies have indicated that the age-dependent decline in episodic memory function starts as early as at the age of 30 (Park et al., 2002), while longitudinal studies propose a later onset between ages 65 and 70 years (Rönnlund et al., 2005). Age-dependent memory decline seems to be paralleled by volume reductions in brain structures important for memory performance, including the medial temporal lobe (Persson et al., 2006), the hippocampus (Rajah et al., 2010), and the prefrontal cortex (Van Petten et al., 2004). Although, it is well known that the amygdala, besides its prominent role in the generation of emotions, is also involved in memory consolidation after fear conditioning (LeDoux, 2007) and emotional events (McIntyre et al., 2003; McGaugh, 2004), its role during age-dependent memory decline has attracted surprisingly fewer research activity.

The role of emotions in the decline of episodic memory function that is seen in the course of physiological aging is still poorly understood. Although emotions are not necessarily an integral component of episodic memory, emotional arousal, or psychosocial stress induced in the laboratory during the encoding of episodic information can facilitate episodic memory consolidation into long-term memory (Wolf, 2012).

It has been also proposed that emotions might be a trigger for episodic memory formation (Libkuman et al., 2004; Dere et al., 2010; Kensinger et al., 2011), play a role in the binding of different features of an event into an integrated episodic memory (Nashiro and Mather, 2011), mediate the self-relevant aspect of an episodic memory (Wheeler et al., 1997) and might also determine their durability in a way that strong emotional activation leads to long-durable episodic memories, while weak emotional activation leads only to short-durable episodic memories (Dere et al., 2010). In line with this proposition it has been found that emotions improve the ability of aged individuals to retrieve more details of an event as well as its context (Kensinger, 2009). There is also evidence suggesting that the processing of emotional stimuli and the physiological arousal mediated by the autonomic system in response to emotionally negative stimuli is compromised in aged individuals (Kaszniak and Menchola, 2012). In this study, we investigated the possibility that aging-induced episodic memory decline might be due to changes in emotionality e.g., in terms of the response to stimuli that have been associated with an emotionally arousing context story.

In order to test the emotionality hypothesis of aging-induced episodic memory impairments, we measured episodic memory in young, middle-aged, and aged participants with a novel paradigm which measures memory for different stimuli, the temporal order of their presentation as well as the spatial locations where they have been presented. This test also measures the ability to establish new episodic memories. We further assessed trait and state anxiety in the 3 groups in order to determine if impairments in episodic memory performance are indeed correlated with decreased levels of trait and/or state anxiety.

Given that depressive symptoms are common in the aged population and are associated with cognitive impairments including working memory deficits (Bornstein et al., 1991; Nebes et al., 2000) we additionally probed whether aging-induced episodic memory decline would be associated with impairments in working memory or depressive symptoms.

## Materials and methods

### Participants

Forty-one healthy adult volunteers (♀ = 22, ♂ = 19) participated in this study. The participants were recruited from University Pierre and Marie Curie Paris 6 students and employees and among healthy relatives of patients attending the geriatric hospital Charles Foix in Ivry-Sur-Seine, France. The participants were aged between 21 and 83 years. They were divided into three groups of young (*N* = 17, mean: 26.76 ± 1.69, age range: 21–45), middle-aged (*N* = 16, mean: 55.31 ± 1.06, age range: 48–62) and aged participants (*N* = 8, mean: 79.13 ± 1.33, age range: 71–83).

None of the participants reported a psychiatric record, history of vascular, psychiatric, neurological, motor or oncologic disease, psychopharmacologic or hormonal therapy or any other health issue that would prohibit their testing. All participants had a corrected to normal vision and audition.

The groups were comparable regarding their socio-demographic and educational background. The study was conducted in accordance with the declaration of Helsinki. Written informed consent was obtained from all participants. All experimental procedures have been approved by the local ethical committee of the University Pierre and Marie Curie Paris 6.

### Experimental design

In a reverse translational approach by Pause et al. (2010) the rationale and principles of the episodic-like memory test for rodents (Dere et al., 2005a,b; Kart-Teke et al., 2006) have been adapted to humans. We developed a paradigm that measures the spatio-temporal memory for emotional and neutral pictures presented on an eight-quadrant computer-screen task (Pause et al., 2010). This test has been further developed to be applicable to aged individuals and patient populations.

#### General procedure

**Figure 1:**
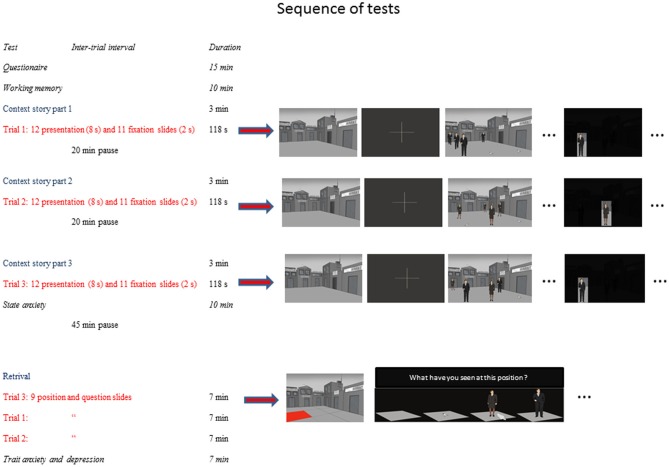
**Experimental design.** Overview of the experimental design, the order of tests applied, presentation times of the slides, and the approximate duration of each experimental phase.

The total experiment including the presentation of general information about the experiment, the completion of the participant questionnaire, the neuropsychological testing as well as the episodic memory test had a total duration of approximately 2.5 h. However, the net time of testing was approximately 65 min. The participants received 3 pauses of 2 × 20 min and 1 × 45 min in the course of the 2.5 h total duration in order to minimize possible effects of tiredness and fatigue on test performance in the older individuals. Figure 1 gives overview of the different phases of the experiment, their sequence, and the approximate duration of each test.

First the participant was informed about the general procedure of the experiment without providing an explicit statement that he or she is participating in a memory experiment or had to retain the information presented during the course of the experiment. It is important to note that no explicit information about the purpose of the test was given. Instead the participants were informed that “The aim of this study is to investigate the effects of imagination on visual perception and attention.” Thereafter, the participants were asked to complete a standard participant questionnaire including questions about the current and past health status, current and past medication, history of mental diseases, etc. After that the participants working memory for series of numbers was tested using a subtest of the WAIS-R. Immediately thereafter the participants performed the episodic memory test consisting of 3 slide presentations followed by an episodic memory test as described in detail below. Immediately after the third slide presentation, the participants were asked to complete the state anxiety subtest of the STAI (Spielberger et al., 1970). Finally, after the participants had completed the episodic memory test, the Goldberg trait anxiety, and depression scale was performed (Goldberg et al., 1988).

### The episodic memory test

This test is based on the “what, where, and when” paradigm (Clayton and Dickinson, 1999; Dere et al., 2005a) that allows to measure the core elements (content, temporal, and spatial context of an unique event) of episodic memory. The present test is designed to measure integrated memories for “what, where, and when” operationalized as “stimulus-position-trial” associations. The test does not allow the assessment of the individual components of episodic memory in terms of content, spatial, and temporal order memory.

**Figure 2:**
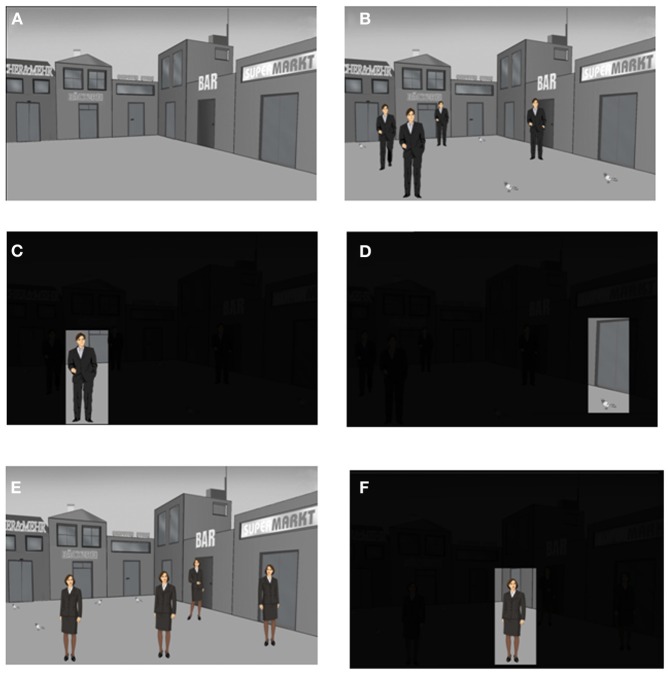
**The episodic memory test: presentation trials 1 and 2.** Each presentation trial is composed of a series of 12 slides. Each slide is presented for 8 s and is followed by the presentation of a fixation cross for 2 s. Schematic drawings of men (presentation trial 1) or woman (presentation trial 2) are used as context story-relevant stimuli while pigeons are used as context story-non relevant stimuli. **(A)** The first slide of each presentation trial shows the background scene without context story-relevant and non-relevant stimuli. **(B)** Presentation of the 8 positions with 4 men as context story-relevant stimuli on the second slide of trial 1. **(C)** Example for the presentation of a single man-position stimulus during the presentation of the slides 3–10 on trial 1. **(D)** Example for the presentation of a single pigeon-position stimulus during the presentation of the slides 3–10 on trials 1 or 2. **(E)** Presentation of the 8 positions with 4 women as context story-relevant stimuli on the second slide of trial 1. **(F)** Example for the presentation of a single women-position stimulus during the presentation of the slides 3–10 on trial 2.

**Figure 3:**
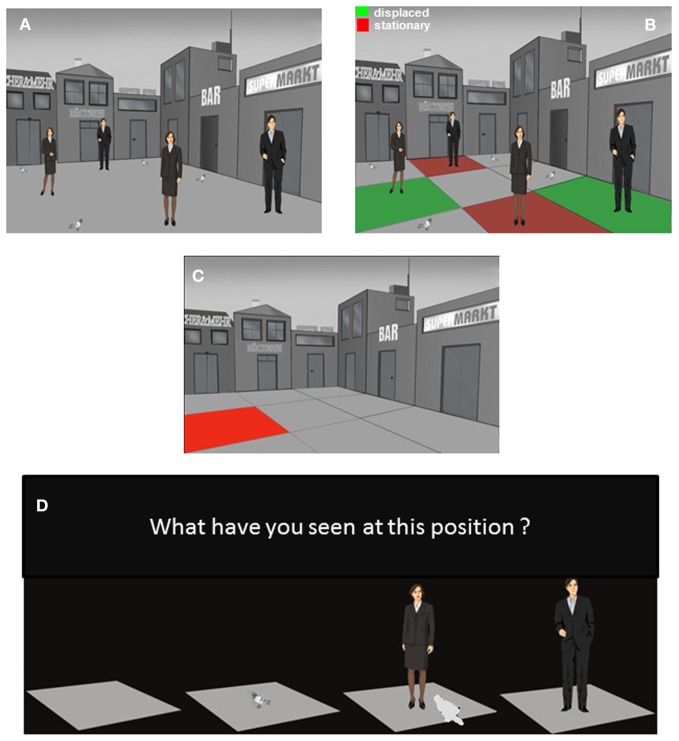
**The episodic memory test: presentation trial 3 and test trial. (A)** Presentation trial 3 where human stimuli from trial 1 and 2 are presented together. Depicted are the 4 positions with human stimuli as context story-relevant stimuli and the 4 position with context story non-relevant stimuli. **(B)** One “old” man and one “recent” woman stimuli were displaced to an unfamiliar position not used during the corresponding presentation trials 1 or 2. Additionally, one “old” men and one “recent” woman stimuli were presented at familiar positions already used during the corresponding presentation trials 1 or 2. The red and green coloring of the quadrants with human stimuli was made for representational purposes and has not been presented to the participants. **(C)** Episodic memory test where participants have to remember the stimulus-position-trial associations formed during the 3 presentation trials. First the positions for the test trial were probed; thereafter the ones for the first trial and finally the ones for the second trial were tested. There were 27 questions in total. On the test trial only the background scene was presented without stimuli. The scene was divided into 9 quadrants. Each of the 9 quadrants was colored and presented individually. **(D)** Thereafter, a slide with the question “What have you seen on this position” and the corresponding response alternatives was presented. The participant was asked to select via mouse click the stimulus which he or she was remembering to be presented on that particular position during a particular presentation trial.

Each participant received 3 presentation trials using the Presentation® software Version 12 (Neurobehavioral Systems, CA, USA) and an episodic memory test (recall of 27 different stimulus-position-trial associations). The 3 trials were embedded into a context story (see below) with emotional content. This context story was divided into 3 parts according to the 3 trials. Each part of the context story was narrated by the experimenter to the participant immediately before the presentation of the corresponding presentation trial. Each presentation trial consisted of 12 slides. Each slide was presented for 8 s and was followed by the presentation of a fixation cross for 2 s. The first slide showed a background scene with an empty place surrounded by shops (Figure 2A). This place was virtually divided into a 3 × 3 matrix with 9 different positions for the presentation of stimuli (Figure 3C). The center position was not used for the presentation of stimuli. The second slide consisted of the same background scene including 4 context story-relevant and 4 context story non-relevant stimuli presented within the 3 × 3 matrix (Figure 2B). Context story-relevant stimuli were drawings of 4 men wearing suits and having slightly different postures (Figure 2B). Stimuli not relevant to the context story consisted of drawings of 4 pigeons (Figure 2B). During the slides 3–10 each stimuli position was presented individually for 8 s while the reminder of the background scene including the other stimuli were darkened (Figures 2C,D). Each slide was followed by the presentation of a gray slide for 2 s showing a plus-shaped fixation cross at the center. This slide ensured the saccadic resetting of the participants view back to the center of the slide before the next individual position was presented. Slide 11 again presented the complete scene including context story-relevant and non-relevant stimuli. This slide was followed by the presentation of the background scene without stimuli. After a delay of 20 min the second presentation trial was performed. This was identical to the first trial, except that drawings of women were presented instead of men (Figures 2E,F). Two of the context story-relevant stimuli (women) were presented at positions already used during the first trial for the placement of context story-relevant stimuli (men), while the remaining 2 context story relevant stimuli were placed at positions which contained context story non-relevant stimuli (pigeons).

After another delay of 20 min the participants viewed the third presentation trial (Figure 3A). The third trial was identical to the first 2 presentation trials except that the 4 context story-relevant stimuli consisted of 2 men (in the following referred to as “old” stimuli with respect to the temporal order of the 2 previous trials) and 2 women “recent” stimuli. Additionally one “old” and one “recent” stimuli were presented at a position which was not used for the placement of context story-relevant stimuli during their initial presentation. Thus one “old” and one “recent” stimuli had been displaced to a “novel” position. These stimuli were referred to as “old displaced” and “recent displaced” stimuli (Figure 3B).

Sixty minutes after the third trial the episodic memory test was performed. Here, the participants were asked to recall each of the 27 stimulus-position-trial associations that have been formed during the 3 presentation trials. The sequence of memory tests for the 3 presentation trials was 3-1-2. First the 9 stimulus-position-trial associations of trial 3 were tested, followed by the tests for trials 1 and finally 2. During the test the participant was presented with the background scene without context story-relevant or non-relevant stimuli. The place was divided into a matrix of 9 positions.

Each episodic memory test for individual trials (1, 2, or 3) consisted of 18 slides. Each individual position, marked with red color, was presented for 2 s (Figure 3C). Thereafter, a slide appeared asking whether the participant remembered to have seen an empty place, a pigeon, a woman, or a man at this position during that particular trial. On the bottom of this slide the 4 choice options were presented as images. The participant could select the right answer by clicking on the corresponding stimuli (Figure 3D). The slide was presented until the participant made a decision. The responses of the participant were automatically recorded and transferred to an Excel® output file. Please note that this procedure allowed the measurement of a memory for what, where, and when in terms of stimulus-position-trial associations with a minimum of verbal requirements allowing the testing of patients with impairments in speech production.

### Context story

The context story was divided into 3 parts which were narrated by the experimenter before each presentation trial and had an emotionally arousing content. Before the experimenter narrated each part of the context story the participant was asked to “Please try to imagine the situation.”

#### Part 1

“Please try to imagine the following situation. You are in a foreign town for 3 days. On the first day you take a walk and reach a small place with little shops and pigeons. You see 4 men in black suits. You recognize these men. You remember to have seen their faces before. They are members of a terrorist group, who had recently attempted to commit a poison gas attack to prevent a political conference. You feel that your heart is beating faster. This attack had been prevented at the very latest moment. If it had been successful, there would have been hundreds of victims. You are quite sure that these men belong to this terrorist group. You now feel cold. You now have goose bumps. You are wondering why these men are here and what they have in mind. You are scared and you think about the cruel and unscrupulous crimes that have been committed by these men in the past.

Then the experimenter asked whether the participant had been able to imagine the situation: “Can you imagine the situation? In the following I will show you the close-ups of the place with the 4 terrorists. When viewing the close-ups please try to imagine what other crimes these people might have committed in the past.”

#### Part 2

“Please try to imagine the following situation.” This is your second day in the foreign town. During a walk you revisit the place with the small shops and the pigeons. This time you encounter 4 women. Again you are sure that you have seen these women before. There was a report on a group of women who have planned a bomb attack to the city hall in the local news this morning. You begin to sweat. This assault could only be stopped seconds before the detonation of the bomb. You are sure that these women are the terrorists who have planned and initiated this assault. Your heart beats faster and your palms are cold. You wonder why these women are here right now. You are scared and you think about the brutal and bloody crimes these women might have committed in the past.

“Can you imagine this situation? In the following I will show you the close-ups of the place with the 4 terrorists. When viewing the close-ups please try to imagine what other crimes these people have committed in the past.”

#### Part 3

“Please try to imagine the following situation”. This is your third day in the town. During a walk you revisit the place with the small shops and the pigeons. This time you see 2 men and 2 woman. You are sure that you have seen these men and woman on the previous occasions. You are breathing faster. You remember to have seen these people in the morning news. It was said that yesterday evening these people have committed an assault to the central station. You remember the pictures of the destruction and the injured victims you saw this morning in the news. While realizing that you are now very close to these dangerous people you are getting horrified. You are feeling sick and your hands are shivering. You feel insecure and you think about the brutal and bloody crimes these people might have committed in the past.

“Can you imagine this situation? In the following I will show you the close-ups of the place with the 4 terrorists. When viewing the close-ups please try to imagine what terrible consequences the attack to the central station might have for the injured innocents.”

### Statistical procedures

Statistical analysis was performed with the program SigmaStat 3.1® (Sysstat Software Inc.). All variables have been initially analyzed with the Kolmogorov–Smirnov test to know whether the data varies significantly from the pattern expected if the data was drawn from a population with a normal distribution. Furthermore, the Levene test was performed to probe the homogeneity of variances across groups. Variables that failed the Kolmogorov–Smirnov or the Levene test were analyzed with nonparametric statistics using the Kruskal–Wallis one-way analysis of variance on ranks and Mann–Whitney rank sum tests for pair-wise multiple comparisons. Variables that passed the normality test were analyzed by means of One-Way ANOVA's and Holm–Sidak and Student *t*-tests for pair-wise multiple comparisons. Correlations between the state and trait anxiety measures and the different episodic memory measures (total score, human stimuli score, pigeon stimuli score, and the scores for the trials 1–3) were performed using both Pearson pair-wise and Spearman-rank correlation procedures in dependence of the distribution of the variables. All *P*-values given are two-tailed, and are considered to be significant when *P* < 0.05 or in the case of the Holm–Sidak tests when the *P*-value obtained was lower than the adjusted *P*-level of significance.

## Results

### Episodic memory performance

#### Total episodic memory score

In this experiment we investigated whether a novel test of episodic memory is suited to detect age-related decrements in episodic memory performance by testing 3 different age groups consisting of young [21–45], middle-aged [48–62], and aged [71–83] healthy adult participants. In order to know whether the groups differ in terms of episodic memory performance we calculated a total episodic memory score that could vary between 0 and 27 correctly remembered stimulus-position-trial associations.

**Figure 4:**
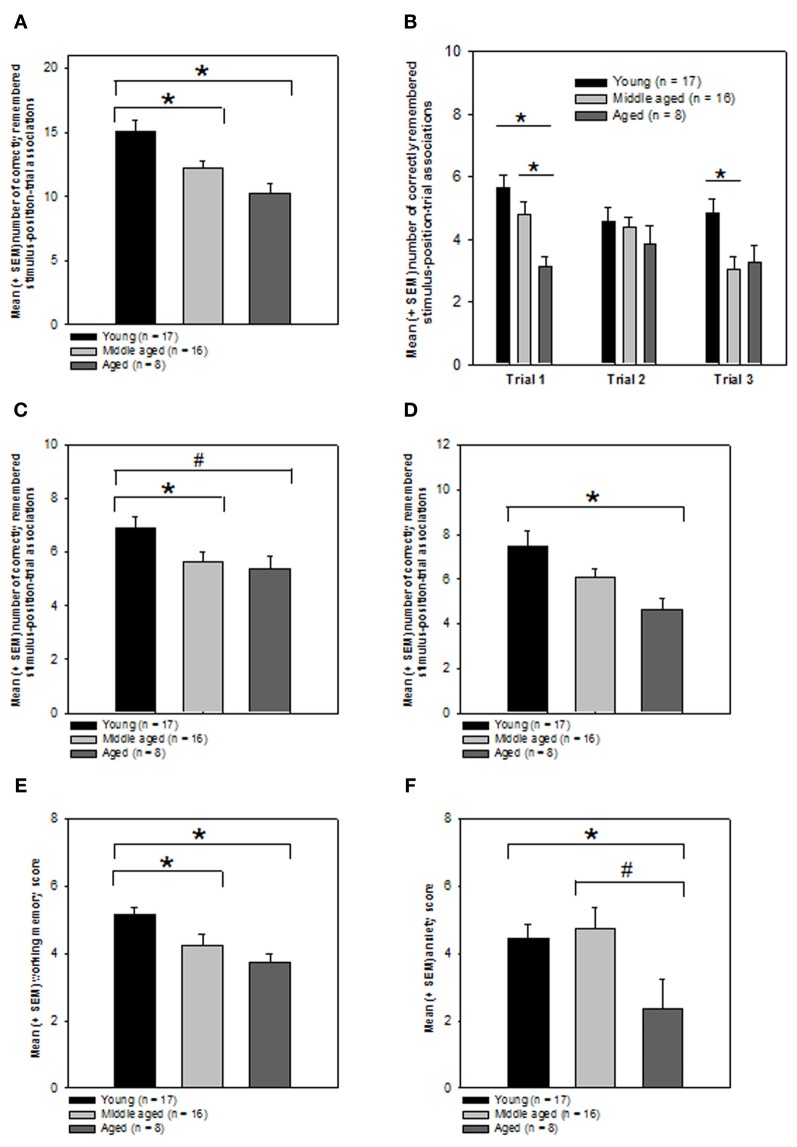
**Effects of aging on episodic memory performance, working memory, and emotionality. (A)** Total episodic memory score. Bars represent mean and SEM number of correctly remembered stimulus-position-trial associations for indicated groups. ^*^P ≤ 0.05, Holm–Sidak test. **(B)** Performance on individual presentation trials. Bars represent mean and SEM number of correctly remembered stimulus-position associations for indicated trials. ^*^P ≤ 0.05, Holm–Sidak test. **(C)** Context story relevant stimulus-position-trial performance. Bars represent mean and SEM total number of correctly remembered human-position-trial associations for indicated groups. ^*^P ≤ 0.05, ^#^0.05 < *P* < 0.1, Mann–Whitney rank sum test. **(D)** Context story non-relevant stimulus-position-trial performance. Bars represent mean and SEM total number of correctly remembered pigeon-position-trial associations for indicated groups. ^*^P ≤ 0.05, Holm–Sidak test. **(E)** Working memory performance. Bars represent mean and SEM working memory scores for indicated groups. ^*^P ≤ 0.05, Mann–Whitney rank sum test. **(F)** Trait anxiety. Bars represent mean and SEM Goldberg anxiety scale scores. ^*^P ≤ 0.05, ^#^0.05 < *P* < 0.1, Student *t*-tests. Graphical presentation of the data: all data obtained with the episodic memory test and the neuropsychological tests were graphically presented as means ± SEM.

As expected a One-Way ANOVA revealed that the performance of the 3 groups was indeed significantly different from each other (Kolmogorov–Smirnov test: *P* > 0.05, Levene test *P* > 0.05; ANOVA: *F*_(2, 38)_ = 7.785, *P* = 0.001, Figure 4A). In order to know which groups performed significantly different from each other, we computed *post-hoc* tests using the Holm–Sidak method. We found that the young group had significantly higher episodic memory scores as compared to both the middle (*T* = 2.675; *P* = 0.011, significant at the critical *P*-level of *P* = 0.025) and aged groups (*T* = 3.72; *P* < 0.001, significant at the critical *P*-level of *P* = 0.017). The latter two groups performed not significantly different from each other (*P* > 0.05).

This result suggest that episodic memory performance is significantly better in young individuals aged between 21 and 45 years as compared to individuals aged between 48 and 62 or older participants.

#### Episodic memory performance for individual presentation trials

We also analyzed whether the groups would differ regarding their episodic memory scores for the three presentation trials. We expected that the episodic memory performance for trial 1 would be significantly different between the groups because the delay between episodic memory encoding and the recollection of the episodic memory formed was longest >2 h for the first trial as compared to the second and third trials. As predicted, we found that the 3 age groups differed significantly regarding the number of correctly remembered stimulus-position associations for trial 1 (Kolmogorov–Smirnov test: *P* > 0.05, Levene test: *P* > 0.05; ANOVA: *F*_(2, 38)_ = 7.056, *P* = 0.002; Figure 4B).

Pair-wise *post-hoc* Holm–Sidak tests revealed that the performance of the aged group during the retrieval of the information for trial 1 was significantly worse as compared to both the young (*T* = 3.756; *P* = 0.000578, significant at the critical *P*-level of *P* = 0.017) and middle-aged group (*T* = 2.489; *P* = 0.0173, significant at the critical *P*-level of *P* = 0.025). No significant difference was obtained for the comparison between the young and middle-aged group (*P* > 0.05).

While no significant difference between the groups was evident for the stimulus-position associations established on trial 2 (Kolmogorov–Smirnov test: *P* > 0.05, Levene test *P* < 0.05; Kruskal–Wallis One-Way analysis of variance on ranks: *P* > 0.05; Figure 4B), the groups performed significantly different when probed for the stimulus-position associations generated on trial 3 (Kolmogorov–Smirnov test: *P* > 0.05; Levene test: *P* > 0.05; ANOVA: *F*_(2, 38)_ = 4.863, *P* = 0.013).

The pair-wise *post-hoc* Holm–Sidak tests indicated a significant difference between the young and middle-aged group (*T* = 2.933; *P* = 0.0057, significant at the critical *P*-level of *P* = 0.017) but not for the remaining 2 comparisons (*P*s > 0.05).

This result suggests that age-related changes in episodic memory performance are reflected by trial 1 and 3 performance. The inability of the second trial to discriminate between the groups might be due to processes of pro- and retroactive interference where the sequential acquisition of learning material has a detrimental effect on the subsequent retrieval process (Brophy et al., 2009). The susceptibility to pro- and retroactive interference might be possibly age-independent.

#### Episodic memory for context-story relevant vs. non-relevant stimuli

Next we analyzed whether the groups might show significant differences in the ability to establish and remember human-position-trial or pigeon-position-trial associations. We expected that the groups would differ significantly for the human context-relevant stimuli but possibly not for the context non-relevant pigeon stimuli.

A One-Way ANOVA on ranks revealed a trend toward a significant difference between the 3 groups regarding the human-position-trial associations (Kolmogorov–Smirnov test: *P* > 0.05, Levene test: *P* < 0.05; Kruskal–Wallis One-Way analysis of variance on ranks: *H* = 5.345, degrees of freedom: 2, *P* = 0.069, Figure 4C). Mann–Whitney rank sum tests for pair-wise comparisons revealed that the young group remembered the human-position-trial combinations significantly better as compared to the middle (*T* = 216.5, *P* = 0.047) group, while there was only a trend for a difference between the young and the aged group (*T* = 74.5, *P* = 0.091). The middle and aged groups performed not significantly different from each other (*P* > 0.05).

Contrarily to our hypothesis, the groups also differed significantly regarding their ability to remember the context-story non-relevant pigeon-position-trial combinations (Kolmogorov–Smirnov test: *P* > 0.05; Levene test *P* > 0.05, ANOVA: *F*_(2, 38)_ = 4.986, *P* = 0.012, Figure 4D). Pair-wise *post-hoc* Holm–Sidak tests for the pigeon-position-trial associations showed that the aged group remembered a significantly fewer number of pigeon-position-trial associations relative to the young group (*T* = 3.075; *P* = 0.00389, significant at the critical *P*-level of *P* = 0.017). There was no significant difference between the young and middle-aged group (*P* > 0.05) or the middle-aged and aged group (*P* > 0.05). Although statistically not significant, the aged group showed slightly better memory performances in the human-position-trial associations compared to the pigeon-position trial condition.

### Within-group comparisons of the episodic memory for context-story relevant vs. non-relevant stimuli

Next we analyzed whether the performance of single groups was significantly different for human vs. pigeon-position-trial associations. However, none of the 3 groups showed a significant difference between the episodic memory for context-story relevant stimuli in comparison to the non-relevant stimuli (all *P*s > 0.05).

### Neuropsychological and psychological assessment

#### Working memory

In order to know whether the expected aging-related differences in episodic memory performance are at least in part due to changes in working memory performance we also measured this capacity in the three groups. A One-Way ANOVA on ranks revealed that the working memory performance of the 3 groups was indeed significantly different from each other (Kolmogorov–Smirnov test: *P* < 0.05; Levene test: *P* > 0.05; Kruskal–Wallis One-Way analysis of variance on ranks: *H* = 13.997, degrees of freedom: 2, *P* < 0.001, Figure 4E). In order to know which groups performed significantly different from each other, we computed Mann–Whitney rank sum tests for pair-wise comparisons. We found that the young group had significantly higher working memory scores as compared to both the middle (*T* = 197.5, *P* = 0.008) and aged groups (*T* = 48, *P* = 0.001). The latter two groups performed not significantly different from each other (*P* > 0.05).

#### Depressive symptoms

It is known that the incidence of major depression is increased in aged individuals and that depressive episodes are associated with memory impairments. We therefore tested whether possible differences in episodic memory performance between the 3 groups might be explained by differences in depressive symptoms. There were no significant differences between the groups on the Goldberg depression scale (*P* > 0.05).

#### Anxiety

We have hypothesized that age-related decline in episodic memory performance might be related to hypo-emotionality or changes in the processing of emotionally-valenced stimuli. Therefore, we also tested whether possible group-differences in episodic memory performance might be explained by differences in trait or state (experimental context-induced) anxiety.

#### Trait anxiety

A One-Way ANOVA indicated a trend for a difference between the groups in the Goldberg scale subtest measuring trait anxiety (Kolmogorov–Smirnov test: *P* > 0.05, Levene test *P* > 0.05; ANOVA: *F*_(2, 37)_ = 2.781, *P* = 0.075, Figure 4F). Pair-wise comparisons by means of Student *t*-tests revealed that the aged group showed significantly lower trait anxiety scores as compared to the young group (Kolmogorov–Smirnov test: *P* > 0.05, Levene test *P* > 0.05; *T* = 2.236, degrees of freedom: 22, *P* = 0.036), but only a trend for a difference as compared to the middle-aged group (Kolmogorov–Smirnov test: *P* > 0.05, Levene test *P* > 0.05; *T* = 2.016, degrees of freedom: 22, *P* = 0.056). No significant difference was found for the comparison between the young and middle-aged group (*P* > 0.05).

#### State anxiety

The comparison of the STAI state anxiety reference values reported for patients with phobia or anxiety disorders (*F*40/*F*41) mean: 42.7 ± 11.5 (Laux et al., 1981) with the mean scores of our 3 age groups: young: 50.59 ± 0.68, middle aged: 52.25 ± and aged: 51.0 ± 2.67 indicates that the context story in combination with the slide presentations has induced an level of emotional arousal in the participants that is similar to the one that has been measured in clinical populations. However, there was no significant difference between groups in the state anxiety test (*P* > 0.05). This result suggest that the experimental context induced similar levels of emotional activation respectively anxiety in the 3 groups.

#### Correlations

We also performed Pearson and Spearman rank correlations including data from all participants between different measures of episodic memory performance and state and trait anxiety scores. There was only one significant correlation between the state anxiety scores and the episodic memory scores for the pigeon-position-trial associations (*R*_(38)_ = 0.322, *P* = 0.049) all other correlations obtained failed to reach the level of statistical significance. We also calculated single group correlations for the significant correlation found between state anxiety and pigeon-position-trial associations, but did not found significant correlations between the two variables in any group (all *P*s > 0.05). The highest correlation found was *R*_(16)_ = 0.431 with a *P*-value of 0.095 in the middle aged group.

## Discussion

### Summary

In the present study, we hypothesized that episodic memory deficits will be associated with changes in trait and/or state anxiety in the aged but also in the middle-aged group. The results presented above suggest that our novel test of episodic memory that measures the core components of an episodic memory (event, spatial, and temporal information) and in addition probes the ability to form new episodic memories is suited to detect age-related impairments in episodic memory performance. The episodic memory deficits observed in the aged group were observed along with lower anxiety scores measured with the Goldberg anxiety scale. However, no significant correlation between episodic memory and trait or state anxiety scores were found.

### Episodic memory performance on individual presentation trials

It is well known that aging affects episodic memory function more severely than other types of memory including semantic memory (Levine et al., 2002). It has been proposed that this difference is possibly mediated by changes in the processing of emotionally-competent stimuli (Allen et al., 2005; Kensinger, 2009). We found that the young group was able to remember a higher number of stimulus-position-trial associations (out of 27 associations to be remembered) as compared to both the middle-aged and aged groups. Age-dependent effects were most prominent when the recall of the information for trial 1 was tested. Here, the aged group performed inferior to both the young and middle-aged groups. These results suggest that the aged group failed to consolidate the trial 1 information (that had been acquired more than 2 h before the test) into long-term memory.

### Context story relevant and non-relevant stimulus-position-trial performance

We hypothesized that context story-relevant stimuli (men and woman) might be generally better remembered as context story non-relevant stimuli and therefore would be much better suited to identify age-dependent impairments in episodic memory performance. However, the present results suggest that this was not the case. In fact there was no significant difference between the memory of the context story-relevant vs. non-relevant stimuli even in the young group. We found that both types of stimuli were suited to detect age-dependent changes in episodic memory performance. As expected the young group showed better performance for context story-relevant stimuli as compared to the other groups. Interestingly, the aged group showed impaired context story non-relevant recall of pigeon-position-trial associations as compared to the young group. The aged group also showed slightly better (although statistically not significant) memory performance in the context-story relevant human-position-trial associations condition as compared to the pigeon-position-trial associations condition. These results suggest the presence of an impairment in the possibly incidental or non-intentional encoding of information that is not directly relevant to the context-story in the aged group (Naveh-Benjamin et al., 2009). It seems that the limited attention and memory capacities of aged individuals do not permit the allocation of additional processing resources to attend to and encode context-story non-relevant stimuli. Another possible explanation of this deficit might be that the episodic memories encoded by aged individuals generally contain less event details and context-specific information. In fact there is evidence for limited information processing capacities in aged individuals as well as changes in the management and allocation of processing resources in learning situations (Craik and Byrd, 1982).

Interestingly we found a significant correlation between the state anxiety scores and the episodic memory scores for the pigeon-position-trial associations. Given that the old group showed lower trait anxiety scores and impaired episodic memory for pigeon-position-trial associations, it is tempting to speculate that incidental or non-intentional encoding of context-story non-relevant information depends on the level of trait anxiety.

### Aging-dependent effects of working memory on the encoding of episodic information

It is known that working memory mediated by the dorsolateral prefrontal cortex plays a role in the encoding and retrieval of long-term memories including episodic memory (Lee et al., 2000; Cabeza et al., 2002; Blumenfeld and Ranganath, 2006). Thus, we also investigated whether age-related differences in episodic memory performance might be explained by changes in working memory performance. We found that working memory performance is impaired in the middle-aged and aged groups as compared to the young group. It is possible that these working memory deficits might be related to an impairment at the encoding stage of episodic memory formation (Unsworth et al., 2011) in the middle-aged and aged groups in the course of the presentation of the 3 trials.

### Depressive symptoms and episodic memory

It is known that the incidence of depressive symptoms that also include episodic memory impairments are increased in the aged population (Airaksinen et al., 2004, 2007; Potter and Steffens, 2007). In geriatric settings cognitive symptoms that are associated with a depressive disorder are often misinterpreted as dementia (Ouldred and Bryant, 2008). Therefore, we tested whether the middle-aged and aged group might exhibit an increase in depressive symptoms. However, this was not the case so that the differences in episodic memory performance observed between the young and the older groups are unlikely to be due to a depressive condition in the middle-aged and/or aged group.

### Aging-related impairments in episodic memory and changes in emotionality

It is well known that emotionally arousing events are more likely to be encoded into long-term memory as compared to neutral events (Cahill and McGaugh, 1998). Furthermore, it has been hypothesized that emotional activation might be a prerequisite for episodic memory formation (Dere et al., 2010). In the present study, we asked whether aging-related episodic memory decline might be associated with changes in state or trait anxiety. Trait anxiety reflects a general personality trait to exhibit anxiety-related behavior at the cognitive (e.g., worrying) and/or behavioral level (e.g., avoidance of anticipated fear-inducing situations) that might be affected by aging. Trait anxiety is relatively stable across time while state anxiety shows large fluctuations and depends on the current context.

We found lower anxiety scores in the aged group as compared to the young and middle-aged group. This change in the trait anxiety levels in the aged group might be associated with a reduced emotional activation by the experimental situation or by the context story. Given that there were no significant correlations between total episodic memory and trait or state anxiety scores, it still remains to be determined whether this difference in anxiety is indeed related to the impaired episodic memory performance in the aged group. In future studies, we will measure physiological correlates of emotional activation such as the blood pressure and the galvanic skin reaction in the aged population to test the above proposed relationship between decreased anxiety and impairments in episodic memory in healthy aged individuals.

There is evidence that older individuals exhibit a decreased functional connectivity between the amygdala and the hippocampus (St Jacques et al., 2009). Episodic memory impairments in the elderly might be related to a diminished emotional modulation of declarative memory formation implemented by the amygdala-hippocampus axis (McGaugh et al., 1996; Tulving and Markowitsch, 1998; Dere et al., 2010). This hypothesis might be tested by combining our novel test of episodic memory with hippocampal-amygdala EEG coherence measurements and neuroimaging methods such as functional MRI.

#### Novel tests of episodic memory and limitations of the present task

Recently novel episodic memory tasks have been designed that are based on paradigms that have been originally developed to study episodic-like memory in animals (Clayton and Dickinson, 1999; Dere et al., 2005a). These tasks are either based on the “what, where, and when” (Pause et al., 2010; Holland and Smulders, 2011) or “what, where, which” (Easton et al., 2012) principles. Pause et al. (2010) followed a reverse translational approach and adapted the episodic-like memory test for rodents (Dere et al., 2005a) to humans and devised a computer-based test. However, this test requiring the active exploration of the computer screen using a keyboard might be too complicated to be run with older individuals. Others have probed episodic memory based on the “what, where, and when” paradigm by asking participants to hide different coin types (what) in different locations (where) on two separate occasions (when) (Holland and Smulders, 2011). These authors conclude that paradigms based on the “what, where, and when” paradigm might be indeed reliable tests of episodic memory function. Another approach to translate findings from animal research into tests of human episodic memory was based on the “what, where, and which” paradigm (Easton et al., 2012). Here, it has been proposed that tasks using contextual information to discriminate events could only be accurately performed using recollection, not familiarity, while tasks using temporal information to discriminate events might be solved using either recollection or familiarity (Easton et al., 2012). It thus remains to be determined whether tasks based on the “what, where, and when” paradigm rely more on recollection vs. familiarity-based memory performance.

In order to fully explore the strengths and limits of the novel episodic memory test described here, follow-up studies should be performed in which recollection vs. familiarity-based memory performance is assessed, sample sizes are increased and in which direct physiological measurements of emotional activation during task performance are correlated with the episodic memory scores obtained.

### Conflict of interest statement

The authors declare that the research was conducted in the absence of any commercial or financial relationships that could be construed as a potential conflict of interest.
